# Multimodal Data Fusion in Learning Analytics: A Systematic Review

**DOI:** 10.3390/s20236856

**Published:** 2020-11-30

**Authors:** Su Mu, Meng Cui, Xiaodi Huang

**Affiliations:** 1 School of Information Technology in Education, South China Normal University, Guangzhou 510631, China; musu@m.scnu.edu.cn; 2 School of Computing and Mathematics, Charles Sturt University, Albury, NSW 2640, Australia; xhuang@csu.edu.au

**Keywords:** multimodal learning analytics, data fusion, multimodal data, learning indicators, online learning

## Abstract

Multimodal learning analytics (MMLA), which has become increasingly popular, can help provide an accurate understanding of learning processes. However, it is still unclear how multimodal data is integrated into MMLA. By following the Preferred Reporting Items for Systematic Reviews and Meta-Analyses (PRISMA) guidelines, this paper systematically surveys 346 articles on MMLA published during the past three years. For this purpose, we first present a conceptual model for reviewing these articles from three dimensions: data types, learning indicators, and data fusion. Based on this model, we then answer the following questions: 1. What types of data and learning indicators are used in MMLA, together with their relationships; and 2. What are the classifications of the data fusion methods in MMLA. Finally, we point out the key stages in data fusion and the future research direction in MMLA. Our main findings from this review are (a) The data in MMLA are classified into digital data, physical data, physiological data, psychometric data, and environment data; (b) The learning indicators are behavior, cognition, emotion, collaboration, and engagement; (c) The relationships between multimodal data and learning indicators are one-to-one, one-to-any, and many-to-one. The complex relationships between multimodal data and learning indicators are the key for data fusion; (d) The main data fusion methods in MMLA are many-to-one, many-to-many and multiple validations among multimodal data; and (e) Multimodal data fusion can be characterized by the multimodality of data, multi-dimension of indicators, and diversity of methods.

## 1. Introduction

Learning analytics refers to the measurement, collection, analysis, and reporting of data about learners and their learning contexts, for understanding and optimizing learning and the environment in which it occurs [1]. The data for traditional learning analytics is usually unidimensional [2]. For example, only log data rather than all data generated by a learning management system are commonly used for analyzing the online learning process. Specifically, log data ignore important contextual information about learners [3]. These context data are crucial for understanding students’ learning processes. In other words, unidimensional data provide only partial information about the learning process [4,5], which makes it impossible to produce accurate results of learning analytics [6]. The real learning process is complex [7]. To understand a learning process accurately [8], we must collect multimodal data such as learning behavior data, facial expression data, and physiological data as much as possible [7]. In this way, a better, more holistic picture of learning can be revealed.

As a new area of learning analytics [7], multimodal learning analytics (MMLA) [9] captures, integrates, and analyzes learning traces from different sources in a way that enables a holistic understanding of a learning process. By leveraging sophisticated machine learning and artificial intelligence techniques [10], MMLA focuses mainly on its paradigms [11,12], framework [6,13], multimodal data [14,15], system [16,17], multimodal data value chain [18], and case studies [19,20]. 

Data fusion is a crucial component in MMLA [21]. Different types of data play different roles in their integration. However, data from different sources are often collected at different grain sizes. This makes it difficult to integrate them [22]. Therefore, it is necessary to review the existing MMLA research to understand the ways multimodal data is integrated. This literature review can help researchers to have a deeper understanding of multimodal data integration and promote the development of related research in this area.

The available reviews on MMLA have been conducted from different perspectives, such as its past, present, and potential futures [10,23,24,25], architectures [26], the multimodal data, and the learning theories in MMLA [7], and MMLA for children [27]. However, to the best of our knowledge, a systematic review with a focus on data fusion in MMLA is not available. To fill this gap, we attempt to present a systematic review of MMLA articles published between 2017–2020, by answering the question of how to integrate and analyze multimodal data. Conducting a detailed review of the research in MMLA, we aim to have a clear understanding of the current research status of multimodal data integration by analyzing the approaches for integrating multimodal data. Through the systematic analysis, we outline the future directions of multimodal data integration. Specifically, the three research questions in this study are as follows:

RQ 1: What is the overall status of MMLA research? (Section 2.4)
RQ 2: What types of multimodal data and learning indicators are used in MMLA? What are the relationships between multimodal data and learning indicators? (Section 5)
RQ 3: How can multimodal data be integrated into MMLA? What are the main methods, key stages, and main features of data fusion in MMLA? (Section 6)

The contributions of this paper are: (1) we propose a novel MMLA framework; (2) according to the proposed framework, this paper summarizes the broad data types and learning indicators in MMLA, proposes a multimodal data classification framework, and characterizes the relationships between multimodal data and learning indicators, and; (3) we review the integration methods and main stages of data integration in MMLA, describing the three-dimensional characteristics of data integration.

The rest of this paper is organized as follows. Section 2 describes our methods and the detailed process of literature review and summarizes the overall research status of MMLA. Section 3 presents the MMLA model. Section 4 outlines the data types, learning indicators, and their relationships in MMLA. Section 5 reviews the data integration methods, main stages in MMLA and points out the future research directions in MMLA. Section 6 concludes the paper. 

## 2. Survey Methods

As a method for systematic review and meta-analysis, Preferred Reporting Items for Systematic Reviews and Meta-Analyses (PRISMA) [28] is commonly used for reporting an evidence-based minimum set of items. Primarily in the context of healthcare, PRISMA provides guidelines that consist of a checklist of 27 items on the title, abstract, methods, results, discussion, and funding, as well as on a four-phase flow diagram. The flow diagram illustrates the systematic review and clearly outlines the study identification, screening, eligibility, and inclusion processes, including reasons for study exclusion.

**Figure 1:**
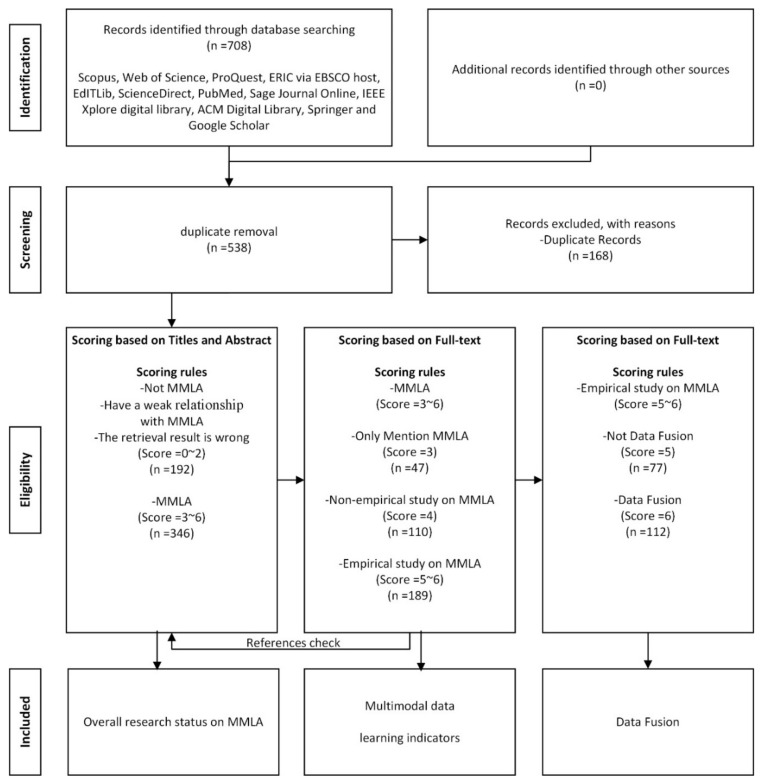
Flow diagram based on the Preferred Reporting Items for Systematic Reviews and Meta-Analyses (PRISMA) guidelines.

Following the PRISMA guidelines [28], we conducted a systematic review on how to integrate multimodal data in MMLA, by using an explicit and replicable search strategy. In particular, we selected the literature on MMLA based on pre-determined criteria, which have been used for other systematic reviews in education research [29,30,31,32,33]. The procedure of our review is illustrated in the flow diagram in Figure 1. First, the relevant articles from the databases were retried, followed by removing duplicate articles. The articles were then scored and coded according to the inclusion and exclusion criteria. Finally, we conducted a detailed analysis of all the included articles by answering the proposed research questions.

### 2.1. Search Method

Using the keywords “Multimodal Learning Analytics”, “MMLA”, “multimodal”, and “Learning Analytics”, we retrieved relevant papers from 12 bibliographic databases. They were Scopus, Web of Science, ProQuest, ERIC via EBSCO host, EdITLib, ScienceDirect, PubMed, Sage Journal Online, IEEE Xplore digital library, ACM Digital Library, Springer, and Google Scholar. Their references to the key retrieved articles were further retrieved for reviewing additional relevant papers. All the articles were limited to publications between January 2017 and June 2020. Additionally, three separate searches were conducted for those published in December 2019, March 2020, and June 2020. In November 2020, we conducted the last round of supplementary searches. As a result, the initial search produced 708 articles. 

### 2.2. Inclusion and Exclusion Criteria

Table 1 shows the inclusion and exclusion criteria for this review. All the reviewed articles have met the inclusion criteria. Finally, 538 articles in total were included after removing duplicate articles.

### 2.3. Scoring and Encoding

After reading its title, abstract, and full text, we scored each of the 538 articles according to the scoring rules listed in Table 2. Highly similar articles, for example, [4,34,35], were treated as the same category by assigning the same score. We excluded articles with their scores below 3 (not including 3) because they have no or little relation to MMLA. In the end, 346 articles on MMLA were included. Articles with different scores were used to answer different research questions. We conducted a detailed analysis of each included empirical study in the paper, identified its multimodal data and learning indicators, and distinguished them by using short notations. For example, the eye movement data were denoted as EM and the electroencephalogram data as EEG. The detailed notations are given in Table 3.

### 2.4. Overall Research Status (Q1)

Table 4 reports the scoring results. From the results, it can be seen that MMLA has focused on theoretical and empirical research. In particular, empirical research on multimodal data fusion accounts for a relatively large proportion of the overall research (37.90%). This indicated that data integration is an important part of MMLA research. There are many existing pieces of research on multimodal data integration, and most of them are empirical studies, aiming to solve a specific problem of multimodal data integration. However, there is currently a lack of a theoretical and overall review of the current research status of multimodal data integration. So, it is necessary to conduct a systematic review of how to integrate the data in MMLA.

## 3. MMLA Conceptual Model

**Figure 2:**
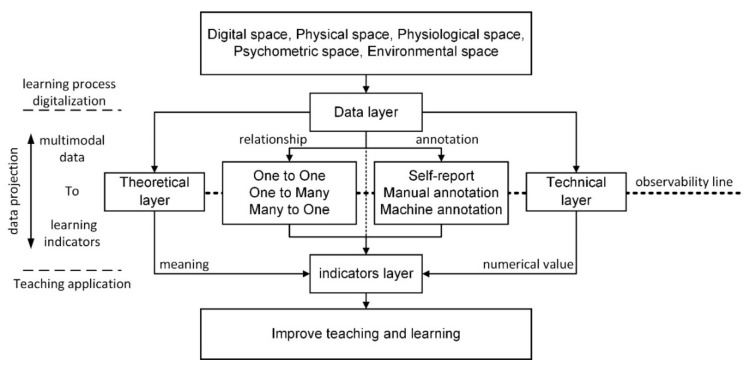
A conceptual model of multimodal data analysis.

Understanding the relationships between multimodal data and learning indicators is essential for MMLA [7]. As shown in Figure 2, we proposed a conceptual model for multimodal data analysis. The purpose of the proposed conceptual model was to better understand the relationships between multimodal data and learning indicators. The conceptual model consisted mainly of three stages and four layers. The three stages were: (a) acquisition of data on the learning process, (b) mapping multimodal data into learning indicators measured, and (c) improvement of students’ learning performance. The three stages focused on external learning behavior, the internal psychological mechanism, as well as practical teaching and learning, respectively. The four layers referred to the data layer, indicator layer, theory layer, and technology layer. The data layer was about data on visible and directly measurable learning behavior, such as eye movement data. The indicator layer represented the invisible learning indicators relating to the sense-making process that cannot be directly measured, such as learning performance, behavior, and emotion. Although the analysis of multimodal data offered a holistic picture of learning, its inherent complexity made it difficult to understand and interpret [74]. Current digital systems are largely blind to users’ cognitive states [55]. There is a conceptual line of demarcation between the data layer and the indicator layer. All observable evidence was above the line, with all the possible interpretations below the line. The semantic interpretation of the data layer was weak in that it could not be used to directly explain the learning process [75]. However, the data layer could be converted into the indicator layer that directly explained the learning process through psychological and educational theories (theoretical layer) and methods (technical layer). The theoretical layer was about psychological and educational theories which tell us how the relationships are drawn between multimodal data and learning indicators [7]. The technical layer was about the methods of how to transform multimodal data into learning indicators. This process is also called “data projection”. 

The three types of annotation methods that transform multimodal data into learning indicators are manual annotation, self-report annotation, and machine annotation [7]. Manual annotation and self-report annotation are commonly used. However, manual annotation is time-consuming and laborious, and self-report annotation is too subjective. Therefore, these two methods are not suitable for large-scale automatic analysis. With the advance of intelligent techniques, automatic machine annotation [35] has received more and more attention. By comparing the accuracies of manual and machine annotations, some studies concluded that a combination of the two methods performs better, producing more accurate results [76,77,78].

The ultimate goal of MMLA is to improve the quality of teaching and learning. The applications of MMLA in teaching and learning mainly include (1) real-time visual feedback of the learning process [79,80,81]; (2) real-time monitoring of the learning process, such as the real-time assessment of attention in the classroom [82], and real-time analysis of teacher-student interactions in a classroom [83], and; (3) teaching design supported by multimodal data, which promotes students’ cognitive development [84].

## 4. Multimodal Data, Learning Indicators and Their Relationships (Q2)

### 4.1. Multimodal Data

**Figure 3:**
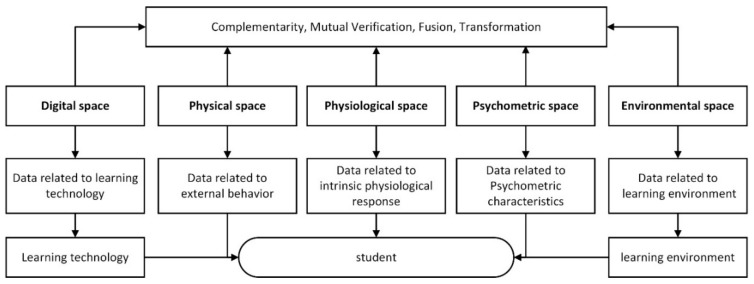
The classification framework of the multimodal data.

Most of the existing MMLA studies recognized the importance of multimodal data. However, few studies systematically classified multimodal data types. As shown in Figure 3, we grouped data used in the existing literature on MMLA [7] into different types in our multimodal data classification framework, together with the typical examples as given in Table 3. 

Specifically, our classification framework consisted of digital space [7], physical space [85], physiological space [71], psychometric space, and environmental space [61]. Digital space referred to various digital traces generated on the system platform during the learning process, such as an online learning platform [52], virtual experiment platform [22], or STEAM educational software [86]. Physical space was about the data obtained by various sensors, such as gesture, posture, and body movement. With the development of sensors, the physical data obtained was more refined at the micro-level, such as the angle of head movement [56] and finger movement on a screen [87]. The perception and analysis of physical data was significant for interpreting the learning process. Physiological space referred to the data related to human internal physiological reflection, including EEG and ECG, which objectively reflected students’ learning status. In contrast, psychometric space, a relatively common source of learning data, referred to various self-report questionnaires that subjectively reflected the learner’s mental state. Environmental space referred to the data about a learning environment where a learner was physically located, such as temperature and weather. Studies have shown that a learning environment has some influence on learning [61]. The increasing analysis of environmental data is a trend in MMLA. Based on this framework, three problems that researchers in MMLA have faced are (1) How to obtain multimodal data, (2) How to use multimodal data to infer students’ learning status (emotions, cognition, attention, etc.), and (3) What learning services can be provided to students based on MMLA?

Due to technological advances such as the Internet of Things, wearable devices, and cloud data storage, learning data at the high-frequency, fine-grained, and micro-level can be collected conveniently and accurately. From multiple dimensions, MMLA reflects learners’ real learning state better [7], especially in some courses [6]. Students interact with learning content, peers, and teachers in a variety of ways, such as facial expressions, audio, and body movements. It is essential that the learning processes are analyzed by using these multimodal data. 

The multimodal data are complementary, mutual verification, fusional, and transformed. (a) Complementarity is an important characteristic of multimodal data. Any data types provide a partial explanation about a certain learning phenomenon or process. (b) Mutual verification–—the same results are verified by different types of learning data [7]. (c) Fusion–—some data integration systems store data in physical spaces such as body movements and gestures in synchronization with log data in digital platforms [7]. (d) Transformation–—physical data are transformed into digital data. Two examples are given below: digitizing students’ handwriting processes through a smartpen and then predicting learning performance through dynamic writing features [41]; digitizing the traces and footnotes of students reviewing a paper test, and then analyzing students’ review behavior [44,88]. The advantage of these studies is that they break the limitations of recording data methods through the use of a mouse and keyboard by retaining the information about students’ authentic learning behavior and learning states as much as possible.

### 4.2. Learning Indicators

The common learning indicators used in the MMLA literature are behavior, attention, cognition [89], metacognition [90], emotion [91], collaboration, interaction [47], engagement, and learning performance. Some of them can be further classified. In particular, learning behavior is divided into three categories—online learning behavior [88], learning behavior in the classroom [53], and embodied learning behavior [92]. Attention includes personal attention [93] and joint attention [45]. Emotions refer to those in autonomous learning [94] and collaborative learning [51]. The collaboration consists of face-to-face collaboration [48] and remote collaboration [95]. Engagement refers to engagement in autonomous learning [52] and the face-to-face classroom [96]. As a summary evaluation, the examination score [59,97], the score of game learning [98], is the common learning performance indicator. Some studies propose complex performance calculation methods to improve the accuracy of learning performance evaluation [99]. Some use formative assessment methods to evaluate learning performance, such as collaborative problem-solving ability [37,56,86,91,100]. Some studies focus on various aspects of learning performance, such as collaboration quality, task performance, and learning [101]. Skills include oral presentation skills [102] and medical operation skills [103].

By examining learning indicators, we found that: (1) There are different kinds of learning indicators, which reflect the complexity of the real learning process; and (2) The meaning of some learning indicators overlapped–the indicators related to learning scene, learning activities, and learning theory. For example, some studies conducted a separate analysis of behavior [44], cognitive engagement [104], and emotion [68] in the learning process. In contrast, some studies combined the three factors to measure learning engagement. Relying on the theory of engagement, Kim et al. [105] observed engagement by using different modalities of linguistic alignment as an indicator of cognitive engagement, kinesics as bodily engagement, and vocal cues as emotional engagement. As another example, collaborations can be analyzed separately [106] and learning engagement in collaborative learning can also be analyzed [105]. (3) There are some rules for selecting learning indicators. Collaborative learning focuses on collaborative features [56] and collaborative interaction [107], while autonomous learning focuses on attention [108], cognition [55], and engagement [39]. There are more learning indicators of face-to-face collaboration [48], with relatively few of remote collaboration [46]. (4) With a more in-depth examination of the learning process, learning indicators will be more diverse. For example, the researchers first paid attention to the learning path in the whole learning process and then focused on the learning path in each webpage from a micro perspective [109].

### 4.3. The Relationships between Multimodal Data and Learning Indicators

MMLA creates a multi-dimensional exploration space to complicate the relationship between data and indicators [24]. The relationships between multimodal data and learning indicators are shown in Table 5. This study found that there were three types of corresponding relationships between multimodal data and learning indicators (multimodal data vs. learning indicators): one-to-one, many-to-one, and one-to-many. “One-to-one” meant that a type of data was suitable only for measuring one learning indicator. This was the most common type in MMLA literature. With the development of technology, the measurement potential of each type of data is gradually tapped. The type of the corresponding relationship, “one-to-one”, has become increasingly rare. For example, the most common methods to measure cognition are interviews and self-reported questionnaires [89]. By means of the new sound-thinking method, cognition is measured by using audio data in the method of thinking aloud [50]. As the physiological measurement is available, physiological data such as EEG are also used to measure cognition [67]. We regarded these new methods as the second type of the corresponding relationship: many-to-one. Precisely, “many-to-one” referred to the fact that multiple types of data measure the same learning indicators. For example, EM, EEG, and EDA measure the degree of learning engagement of learners [110]. Finally, “one-to-many” is the third type of the corresponding relationship; that is, one type of data measures several types of learning indicators. For example, eye movement data measures attention [93], cognition [84], emotion [111], collaboration [46], and engagement [83].

The underlying reason why there are diverse corresponding relationships between learning data and learning indicators is that the range of valid measurement and quality of learning data vary with technical and theoretical conditions. In general, the measurement range of a particular type of data is limited, with obvious advantages in terms of its measurement. There are one or several learning indicators with better measurement effects. For example, online learning data (e.g., logs) are often used to characterize learning behavior [88], while eye movement data are often used to analyze a learner’s cognitive state, attention level, and information processing process about learning content [133]. The facial expression has a better measurement effect on emotion [68] and engagement [83]. Facial expression is a good measure of strong emotion (joy and anger), and physiological data on subtle emotion [134]. Studies have shown that a learning indicator can be measured using either single-dimensional or multi-dimensional data. The measurement of learning indicators must consider not only the optimal data but also the supplement of other types of data, which is of significance to data fusion.

## 5. Data Fusion (Q3)

We analyzed the empirical studies on multimodal data fusion from three aspects: integration methods, data type, and learning indicators. The results are reported in Table 6.

**Figure 4:**
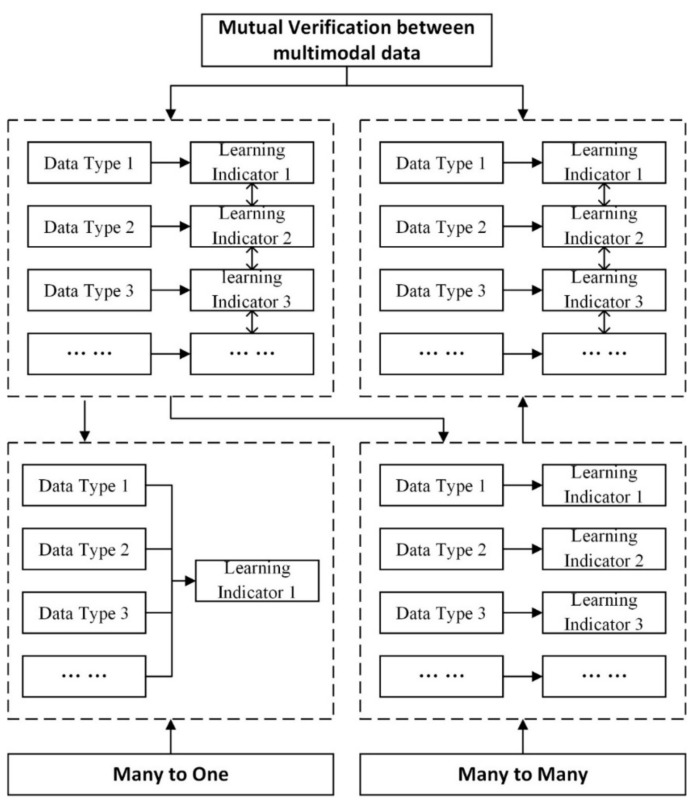
Data integration methods.

According to the four types of multimodal data proposed in this paper, methods for data integration included cross-type data, such as the integration of digital data and physical data [146] and the integration of psychometric data and physiological data [97]. There was also a non-cross-type, such as internal data integration of physiological data types [71,104]. In terms of learning indicators, the current literature on data integration focused on a single indicator, such as learning engagement [83,141], as well as on multiple indicators, such as collaboration, engagement, and learning performance [78,130,138]. From the perspective of the relationships between data integration and learning indicators, data integration can be divided roughly into three categories, as shown in Figure 4: (1) “many to one” (multimodal data vs. learning indicator, for improving the accuracy of measurement), (2) “many to many” (multimodal data vs. multiple learning indicators, for improving information richness), and (3) mutual verification between multimodal data (providing empirical evidence for data fusion and integration). Further, the meaning of data integration in the literature had a broad sense and narrow sense. In a broad sense, the results of experiments on multimodal data were better than on single-mode data. The added value of data integration lies in improving measurement accuracy and information richness, or bringing more meaningful conclusions. In a narrow sense, only “many to one” can achieve data integration.

### 5.1. Integration Methods

#### 5.1.1. “Many-to-One” (Improving Measurement Accuracy)

The characteristics of this category are as follows: (1) There is a clear data integration algorithm model. Multimodal data is usually used as the model input, while one learning indicator is the model output; (2) data integration improves the accuracy of learning indicator measurement. For example, audio data measure emotions [121], and facial expression data also measure emotions [51]. Audio and facial expression data was integrated by [79] to measure emotions and improve the accuracy of emotion measurement. In this line of research, the increase of data mode, the selection of data features, the division of data integration proportion, and the selection of the algorithm will affect the accuracy of the measurement. Some studies have compared single-mode data with multimodal data. The results showed that the measurement from multimodal data integration is more accurate than a single type of data [121,137]. Selecting the features from the raw data that are relevant to learning can increase their interpretability. Some studies just make use of the raw data [68]. In most studies, the data integration ratio is 1:1. As mentioned before, different types of data have different accuracies in measuring the same learning indicator. For example, the use of EM and EEG results in different accuracies in predicting emotion [111]. Therefore, data integration is not as simple as one-to-one mapping. Based on the possible measurement accuracy of various types of data, and the correlations between data and learning indicators, the weights of data types used in the experiments should be allocated accordingly. Finding an efficient algorithm model is key [68]. Machine learning is widely used as algorithm models. Most studies compare the performance of several different algorithm models to determine the optimal algorithm. For example, deep learning methods are compared with traditional machine learning in terms of their performance [37].

#### 5.1.2. “Many-to-Many” (Improving Information Richness)

The characteristics of this type are described as follows: (1) There are more than two multi-dimensional learning indicators; (2) data and learning indicators are the one-to-one mapping; (3) there are no data integration algorithms, and; (4) data integration improves information richness. For example, EM for measuring attention and EEG for measuring cognition are used simultaneously [172]. Multi-dimensional learning indicators can accurately reflect the learning process. So, this line of research needs multiple learning indicators and obtains multimodal data that are suitable for measuring these indicators with the help of data integration systems. Integrated systems include the Oral Presentation Training System [155,156,157], the Sensor-Based Calligraphy Trainer [129], the Medical Operation Training System [103,167], the Ubiquitous Learning Analysis System [168,169], the Classroom Behavior Monitoring System [54,192], and the Dance Training System [115]. Some studies also use one type of data to measure several learning indicators simultaneously. For example, three learning indicators are measured simultaneously by EM (attention, anticipation, fatigue). EEG data are used to measure three learning indicators cload, mental workload, load on memory) [175]. However, this study does not advocate using only multimodal data to measure multiple indicators simultaneously. The overuse of one type of data will also reduce the accuracy of the measurement result to a certain extent. It is necessary to use the most suitable type of data to measure the most suitable learning indicators.

#### 5.1.3. Multimodal Data Validation (Provides Empirical Evidence for Data Fusion)

The objective of this type of MMLA is to increase confidence in the findings by using multimodal data validation, which is also called triangulation. In other words, this type of MMLA produces reliable conclusions through the triangular evidence of multimodal data analysis. Different types of data in an experiment are independent and parallel. Each measures the same learning indicator with different measurement accuracies. Through comparative analysis, we can take the measurement advantages of single-mode data and provide multiple validations for data integration of “many-to-one” and “many-to-many”. For example, [122] first collected multimodal data for collaborative learning analytics. Each type of data measurement collaboration is then analyzed separately, such as audio data [180], body posture [106], movement data [181], and physiological data [101,182]. As another example, self-report data and eye movement data on learning engagement are analyzed [129]. Additionally, some research focuses on the analysis of the relationships between various types of data [128]. The typical questions are what is the relationship between physiological arousal and learning interaction in collaborative learning, when does physiological arousal occur, and how do students’ emotions change [107,128]. Studies by [116,183] used gestures to analyze movement patterns, eye movements to analyze attention patterns, and they analyzed the correlation between movement patterns and attention patterns. Self-report and physiological data are used to measure the cognitive load by calculating the correlation between the two measurements [89,185].

#### 5.1.4. Other Integration Methods

The above three are common data integration methods. With more research on MMLA, methods of data integration will be more diverse in the future. For example, from the perspective of learning process analysis, according to different research questions, selecting different types of data for analysis at different analysis stages is also an idea of data integration. For instance, this multi-step approach uses coarse-grain temporality—learning trajectories across knowledge components—to identify and further explore “focal” moments worthy of more fine-grain, context-rich analysis [22]. As another example, the log data is first used to analyze the overall learning path, and the macro learning path rules are found. Then, the eye movement data is used to analyze the two key learning stages of watching the video and doing testing, to deeply mine the learners’ cognitive preferences.

### 5.2. Summary of the Key Stages of Data Integration and Research Directions

The collection of different fine-grained and multimodal data is the premise of data integration. Within a data synchronous acquisition system, multimodal data on the learning process can be collected at the same time. A data integration system often consists of multiple modules, such as an expression analysis module [83,116,141,183], a VR module [156], a body posture module [142,193], and a self-reflection module [157]. The multimodal data is collected separately first and then co-located by using their timestamps. As a set of tools, STREAMS (Structured TRansactional Event Analysis of Multimodal Streams) integrates log data into multimodal data streams for analyses [22], for example. Therefore, temporal alignment is one of the key steps in data integration.

Data integration analysis is a crucial step in MMLA. Data from different sources are often collected at different times with varying grain sizes. It is highly time-consuming [22] to integrate them. For example, some studies have used data integration acquisition systems, such as presentation training systems, named the Presentation Trainer [155,156,157], but only select a single type of data for analysis. Therefore, these studies are not involved in data integration.

**Figure 5:**
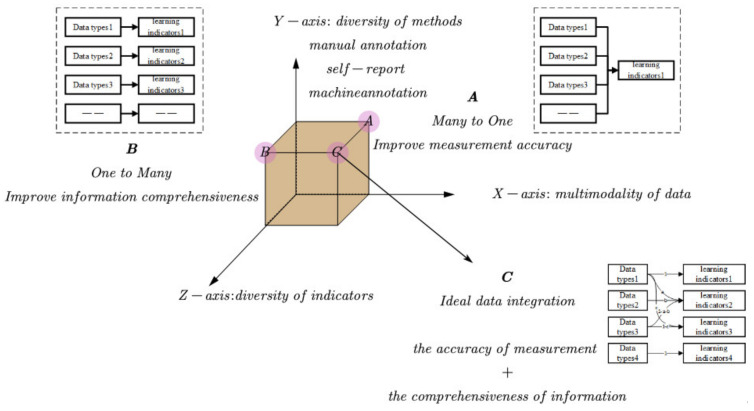
Three-dimensional features of data integration in MMLA.

We summarize MMLA in Figure 5, in which the X-axis represents the multimodality of data, the Y-axis the methods used, and the Z-axis the multidimensional indicators. The existing methods for data integration improve either the accuracy of measure (point A) or the richness of learning information (point B). Ideal data integration should consider the intersection of the X-axis, Y-axis, and Z-axis, such as the C-point. That is, data integration should improve measurement accuracy and information richness, capture the states of learners over their learning, and characterize all aspects of the learning process. For example, the use of eye movement data and log data is measured for cognition, facial expression data for emotions, interview data for metacognition, and self-report data for motivation [94]. In other words, we should make the best use of multimodal data by taking advantage of the individual strengths of its components.

MMLA focuses on what types of data are collected and how to integrate them in a way that the learning process can be characterized accurately. Three factors have contributed to the rapid development of MMLA. First, from the data perspective, the availability of different kinds of perceptual devices that are capable of collecting rich learning data promotes MMLA. Second, from the indicator perspective, the educational inquiry into the mechanism of learning and psychological factors in learning motivates MMLA. Finally, from the method level, the recent advancement of artificial intelligence enables MMLA. 

The use of multimodal data does not mean data integration. Seamless, effective integration of multimodal data for accurately measuring the effectiveness of teaching and learning is an important future research direction of MMLA. Specifically, we believe there are two directions at methodological and practical levels. 

At the methodological level, the research indirections in MMLA may lie in answering the following question. (1) For measuring a given learning indicator, which type of data is well suitable? Some findings on this have already been reported in the literature. However, there is no comprehensive research on answering this question by comparing different types of data against the measurement of learning indicators. (2) How to align multimodal data in a way that the learning indicators can be well-reflected. By experiments, different approaches should be compared in terms of their capacities of capturing hidden correlation information among the data. The complementary information from the different types of data should be exploited by using the similarity-based alignment, for example. (3) For qualifying a given learning indicator, how to fuse the multimodal data so that the complementary correlations from both the intermodality and cross-modality are effectively integrated. A combination of different ways of fusions and the degree of contributions from different types of data to the final performance should be examined.

At the practical level, we should consider how to select the use of multimodal data, data integration degree, and learning indicators, based on the research results. This will involve multi-disciplines such as data science, computer science, and educational technology. The collection and storage of high-frequency, fine-grained, and micro-level multimodal data should be part of a multimodal data education system. Guidance on how to effectively use multimodal data in learning and teaching for educators is a research direction.

In the future, we believe that MMLA will be available in classrooms in real-time.

## 6. Conclusions

As more and more data on learning processes become available, MMLA is becoming increasingly important. This paper has conducted a systematic review of the literature on MMLA published in the past three years. Specifically, we have presented a novel conceptual model for better understanding and classifying multimodal data, learning indicators, and their relationships. We classified the types of multimodal data in MMLA into digital data, physical data, physiological data, psychometric data, and environment data. The learning indicators were grouped as behavior, cognition, emotion, collaboration, and engagement. The relationships between multimodal data and learning indicators were one-to-one, one-to-many, and many-to-one. The complex relationships between multimodal data and learning indicators were the key to data fusion. We summarized the integration methods of multimodal data as many-to-one (improving measurement accuracy), many-to-many (improving information richness), and multimodal data validation (providing empirical evidence for data fusion and integration). Data integration in MMLA has been characterized by three aspects: multimodality of data, multi-dimension of indicators, and diversity of methods. This review highlights that the temporal alignment of multimodal data is the key step in data fusion. A description of three-dimensional characteristics in MMLA was presented and we pointed out the future direction of data fusion in MMLA.

## Figures and Tables

**Table 1:** Inclusion and Exclusion Criteria for Reviewing Papers.

Inclusion Criteria	Exclusion Criteria
The following search keywords are included in the title, abstract, or keywords	Studies published before 2017
“Multimodal Learning Analytics” OR	Duplicate papers (only one paper included)
“MMLA” OR	Articles unrelated to MMLA content
“Learning analytics” and “multimodal”	Non-English papers
	Not Peer-Reviewed

**Table 2:** Scoring rules.

	Scoring Rules			Score	RQ
Title and Abstract	The topic has nothing with MMLA			(Score = 0)	
	The topic has a little with MMLA			(Score = 1~2)	
	MMLA			(Score = 3~6)	Q1 & Q2 & Q3
Full-text		3.1 Only mentioned MMLA		(Score = 3)	Q1
		3.2 Non-empirical study on MMLA, such as its review and theory		(Score = 4)	Q1
		3.3 An empirical study on MMLA		(Score = 5~6)	Q2
			Without Data Fusion	(Score = 5)	
			With Data Fusion	(Score = 6)	Q3

**Table 3:** Multimodal data classification and case studies.

Type	Multimodal Data and Code	Case Studies	Author
**Digital space**			
Clickstream	Log data LOG	Log data as a proxy measure of student engagement	[36]
		Interactions in STEAM by a physical computing platform	[37]
	Mouse MO	Behavioral engagement detection of students	[38]
	Keystrokes KS	Surrogate measure for the effort put in by the student	[39]
Qualitative data	Text TE	Learners’ emotions from pedagogical texts	[40]
	Handwriting Dynamic Handwriting Signal Features HW	Dynamic handwriting signal to predict domain expertise	[41]
		A sensitive measure of handwriting performance	[42]
	Digital footnote DF	Analyzing students’ reviewing behavior	[43,44]
**Physical space**			
Eye	Eye movement EM	Students/teacher co-attention (i.e., with-me-ness)	[45]
		Improving communication between pair programmers	[46]
	Eye Contact EC	Joint Visual Attention	[47]
		Eye contact in three-party conversations	[48]
Mouth	Audio AU	Exploring collaborative writing of user stories	[49]
		Think-aloud protocols used in cognitive and metacognitive activities	[50]
Face	Facial Expression FE	Investigating emotional variation during interaction	[51]
		Automated detection of engagement	[52]
	Facial Region FR	behaviors of lecturers and students	[53]
		Student behavior monitoring systems	[54]
	facial temperature FT	Assess the effect of different levels of cognitive load on facial temperature	[55]
Head	Head Region HER	behavioral engagement detection of students	[38]
		Modeling collaborative problem-solving competence	[56]
Hand	Hand HA	data glove which captures pressure sensitivity designed to provide feedback for palpation tasks	[57]
		Using hand motion to understand embodied mathematical learning	[58]
Arms	Arms AR	Dynamic adaptive gesturing predicts domain expertise in mathematics	[59]
		Embodied learning behavior in the mathematics curriculum	[60]
Leg	step count SC	Step counts are used to predicting learning performance in ubiquitous learning	[61]
Body	Body posture BL	Enhancing multimodal learning through personalized gesture recognition	[62]
		Embodied strategies in the teaching and learning of science	[63]
	Body Movement and Location MP	Making spatial pedagogy visible using positioning sensors	[64]
		Tracing students’ physical movement during practice-based learning	[65]
	Orientation OR	Aggregating positioning and orientation in the visualization of classroom proxemics	[66]
**Physiological space**			
Brain	Electroencephalogram EEG	Detecting cognitive load using EEG during learning	[67]
		Multimodal emotion recognition	[68]
Skin	electrodermal activity EDA	Profiling sympathetic arousal in a physics course	[69]
	galvanic skin response GSR	The difficulty of learning materials	[70]
	skin temperature ST	Recognition of emotions	[71]
Heart	Electrocardiogram ECG	EDA and ECG study of pair-programming in a classroom environment	[72]
		Multimodal emotion recognition	[68]
	Photoplethysmography PPG	Recognition of emotions	[73]
	heart rate /variability HR/HRV	Automated detection of engagement	[52]
Blood	blood volume pulse BVP	Recognition of emotions	[71]
Lung	Breathe respiration BR	Recognition of emotions	[71]
**Psychometric space**			
	Motivation PS	Motivation coming from the questionnaire	[45]
**Environmental space**			
	Weather condition WC	Predicting performance in self-regulated learning using multimodal data, such as (1) Temperature, (2) Pressure, (3) Precipitation, (4) Weather type	[61]

**Table 4:** Scoring results.

Score	Num. of Articles	Percentage	Remarks
3	47	3.36%	Only Mention MMLA
4	110	35.26%	Non-empirical study on MMLA
5	77	24.68%	An empirical study on MMLA BUT without Data Fusion
6	112	37.90%	An empirical study on MMLA AND Data Fusion

**Table 5:** The relationships between multimodal data and learning indicators.

	Learning Indicator	Behavior	Attention	Cognition Metacognition	Emotion	Collaboration	Engagement	Learning Performance
Multimodal Data								
Digital space		[41] [43,44]		[112]	[40]	[95] [113]	[36] [38] [39]	[61] [98] [114] [37] [41]
Physical space		[53] [115] [116] [92] [42] [62] [63] [103] [117] [53] [64]	[93] [118] [108] [119] [82] [45] [120]	[84] [112] [50] [90]	[73] [111] [121] [51] [79] [68]	[122] [47] [48] [123] [49] [124] [125] [126] [91] [127] [107] [65] [128] [95] [113] [46]	[52] [110] [38] [129] [130] [83] [38] [60]	[70] [98] [37] [98] [114] [37] [59] [61]
Physiological space				[67] [69] [131] [89]	[111] [68] [71] [73] [68]	[72] [107] [128] [122] [131] [72]	[52] [110] [130]	[132] [98] [70] [61]
Physiological space			[99] [70]	[89]	[121] [79]		[70] [129] [52]	[61]

**Table 6:** Data integration in multimodal learning analytics (MMLA).

Integration Methods	Data Type	Learning Indicators	Author
Many-to-One	FE, PPG	Emotion	[73,134,135]
	AU, FA, LOG, HA	Learning performance	[37,86,100]
	LOG, AU, BL, SR	Collaboration	[114,136]
	PS, AU	Emotion	[121,137]
	AU, FE, BL, EDA, VO	Collaboration, engagement, learning performance	[78,130,138]
	EM, AU, VB, MP	Teaching behavior	[139,140]
	FE, HER, EM	Engagement	[83,141]
	FR, HER, BL	Engagement	[99,142]
	AR, HER, FR	Collaboration	[56,76,91]
	FE, EM, EEG, EDA, BVP, HR, TEMP	Engagement	[110,143]
	AU, LOG	Collaboration	[113,144]
	AU, LOG	Emotion	[145]
	AU, VB	Engagement	[105]
	FR, MO, LOG	Engagement	[146]
	FE, HR, LBP-TOP	Engagement	[52]
	AU, LOG, BL	Oral presentations	[147]
	PS, AU, FE	Emotion	[79]
	EM, EEG	Emotion	[111]
	AU, FE, ECG, EDA	Emotion	[68]
	VB, LOG	Cognition	[74]
	FE, HER, LOG	Engagement	[96]
	SC, LOG, HR, EN	Learning performance	[61]
	HER, LOG	Engagement	[148]
	PE, PS, AU, FE, BL, EM, EEG, BVP, GSA	Learning performance	[98]
	GSR, ST, HR, HRV, PD	Cognitive load	[149]
	AU, EM, LOG	Dialogue failure in human-computer interaction	[150]
	AU, HAR, FR	Collaboration	[151]
	HAR, EC, FR	Engagement	[152]
	BL, MP, LOG	Attention	[119]
	AU, FE, EM, LOG	Collaboration	[95]
	EEG, EOG, ST, GSR, BVP	Emotion	[71]
	AU, EC, AR, MP	Oral presentations	[153]
	AU, BL, LOG	Embodied learning behavior	[154]
Many-to-Many	FE, BL, AU, EC	Oral presentations	[155,156,157]
	BL, AU	Collaboration	[81,158,159,160]
	MP, AU, LOG, EDA, PS	Medical operation skills	[80,161,162,163,164,165,166]
	BL, EMG, LOG	Medical operation skills	[103,167]
	AU, EM, MP, BL	Embodied learning behavior	[168,169]
	FA, EC, MP	Face-to-face classroom	[54]
	AU, HER, HA, AR, MP	Oral presentations	[102]
	FE, HER, AR, LE, MP	Dancing skills	[115]
	FA, EDA, HR	-	[170]
	AU, MP, BL, LOG	Oral presentations	[171]
	EM, EEG	Attention, cognition	[172]
	-	Dancing skills	[173]
	AU, BL, MP, LOG	-	[174]
	EM, EEG	Adaptive self-assessment activity	[175]
	AU, VB, LOG	-	[176]
	EM, LOG	Open-ended learning environments	[94]
	BL, EC, AU, LOG	Oral presentations	[177]
	MP, FE, AU	Oral presentations	[178]
Mutual Verification between multimodal data	VO, FE, EDA	Collaboration, emotion	[107,128]
	BL,EDA,EM,AU, BVP, IBI, EDA,HR	Collaboration	[101,106,122,179,180,181,182]
	LOG, SR, AU	Online learning	[3,22]
	FR, EC	Embodied learning behavior	[116,183]
	PS, EM, LOG	Calligraphy training	[129,184]
	PS, GSR, ST, LOG	Online learning problem solving	[89,185]
	BL, MP, AU	Collaboration	[127]
	HER, AR	Language learning	[92]
	EDA, ECG	Collaboration	[72]
	EEG, LOG	Cognition	[186]
	-	Collaboration	[187]
	MP, OR	Teaching behavior	[66]
	VB, ONLINE	Emotion	[188]
	EM, BL	Collaboration	[189]
	EDA, PS	-	[97]
	EC, MP	Collaboration	[123]
	FE, EM, GSR	Learning performance	[70]
	EM, FA, LOG	Learning difficulties	[190]
	EM, LOG	Cognition	[112]
	EM, AU, LOG	Engagement, collaboration, learning performance	[191]
